# Treatment of traumatic subluxation of the crystalline lens with custom-made capsular hook

**DOI:** 10.3389/fmed.2025.1569767

**Published:** 2025-03-25

**Authors:** Ning Li, Jie Dong, Jianfeng Wang, Xiaohui Ren, Juanjuan Guo, Juan Li, Ziqing Gao

**Affiliations:** ^1^Department of Ophthalmology, The First Affiliated Hospital of Bengbu Medical University, Bengbu, China; ^2^Department of Ophthalmology, Shenyang Fourth People’s Hospital, Shenyang, China

**Keywords:** zonules, lens dislocation, traumatic cataract, capsular hook, capsular tension ring

## Abstract

**Objective:**

Traumatic rupture of the lens zonules, leading to lens dislocation, is common in clinical practice and often requires surgical treatment. We aim to study capsular hooks, formed from 5-0 or 6-0 polypropylene sutures by heat shaping, to fix the capsular bag and reshape the lens zonular.

**Methods:**

A retrospective analysis was conducted on 16 patients (16 eyes) with traumatic subluxation of the crystalline lens who visited our department. Capsular hooks were fabricated using 5-0 or 6-0 polypropylene sutures, shape by heat, to fix the anterior capsular opening. The sutures passed out of the eye, punctured the sclera and traversed it parallel to the limbus for 2–3 mm, exiting through the conjunctiva to form a suture tunnel parallel to the limbus, securing the suture and preventing loosening. Changes in best-corrected visual acuity (BCVA) after surgery, intraocular lens centration, and surgical complications were assessed.

**Results:**

Post-operative follow-up was 6–30 months, with an average of 10 ± 5.9 months. The BCVA improved from a pre-operative median of 2.15 (1.67) logMAR to a postoperative median of 0.25 (0.38) logMAR (paired sample Wilcoxon signed-rank test, *Z* = 3.516, *p* < 0.001). Postoperatively, the intraocular lenses were well-centered, the capsular bags were securely fixed, and no capsular hooks dislodgement occurred. One patient experienced combined vitreous hemorrhage during surgery. Another patient (case 2) developed rhegmatogenous retinal detachment 2 years post-surgery. After pars plana vitrectomy, the retina was reattached, and the final visual acuity remained stable.

**Conclusion:**

Long-term follow-up data suggest that using custom-made capsular hooks offers a reliable solution for maintaining long-term stability of the capsular bag in patients with traumatic crystalline lens subluxation. This technique maintains the centering of the IOL postoperatively, showing effectiveness and safety, and it holds potential for broader clinical application.

## 1 Introduction

The zonules of the lens are crucial for maintaining its normal position (1). Congenital or acquired factors can damage the zonules, resulting in lens dislocation, a condition characterized by abnormal lens positioning (2). Crystalline lens dislocation is a frequently encountered ophthalmological condition. Zonular defects are commonly seen in congenital conditions, such as Marfan syndrome (3–5), spherophakia (6), and homocystinuria (2, 7). Traumatic rupture of the crystalline lens zonules, leading to lens dislocation, is also common in clinical practice and often requires surgical treatment (8, 9).

Clinically, lens dislocation is often classified into mild (<4 clock hours), moderate (4–8 clock hours), and severe (>8 clock hours) based on the extent of zonular rupture (10). For mild subluxation of the lens, the combination of a standard capsular tension ring (CTR) and intraocular lens (IOL) implantation in the capsular bag can effectively solve the problem of centering the IOL (11). However, for moderate and severe subluxation of the lens, surgical challenges increase. Some patients may not be candidates for IOL implantation in a single procedure, often requiring staged lens extraction followed by suspended IOL implantation (12, 13).

With advancements in surgical techniques and evolving treatment approaches, we have abandoned the traditional surgical method in the treatment of traumatic subluxation of the crystalline lens. Instead, we should try our best to preserve the patient’s capsular bag and reshape the lens zonular. In the absence of commercial capsular fixation devices, and for the purpose of solving the long-term fixation problem of the capsular bag, we used custom-made capsular hooks, formed from 5-0 or 6-0 polypropylene sutures by heat shaping, to fix the capsular bag. This approach has been applied to 16 patients with traumatic crystalline lens subluxation, yielding favorable outcomes. This technique can also be used for congenital subluxation of the lens and lens dislocation caused by other zonular lesions.

## 2 Materials and methods

### 2.1 Patient eligibility

In this study, a retrospective analysis was conducted on 16 patients (16 eyes) with traumatic crystalline lens subluxation who were treated at the Department of Ophthalmology, The First Affiliated Hospital of Bengbu Medical University from April 2021 to August 2024. Eligible patients had a history of ocular trauma and were diagnosed with lens subluxation through slit-lamp microscope examination after pupil dilation. Exclusion criteria included congenital lens subluxation, active infectious ocular conditions, and sever vitreoretional complications, such as retinal detachment, subreinal hemorrhage, etc. Before the operation, the patients were fully informed about the surgical procedure and signed the informed consent forms. All procedures were conducted following the principles of the 1964 Declaration of Helsinki and its subsequent amendments. Approved by the Hospital Ethics Committee (2022033), this study introduced a new surgical technology. Patients were followed up at 6–30 months postoperatively, with an average of 10 ± 5.9 months.

### 2.2 Surgical technique

**Figure 1:**
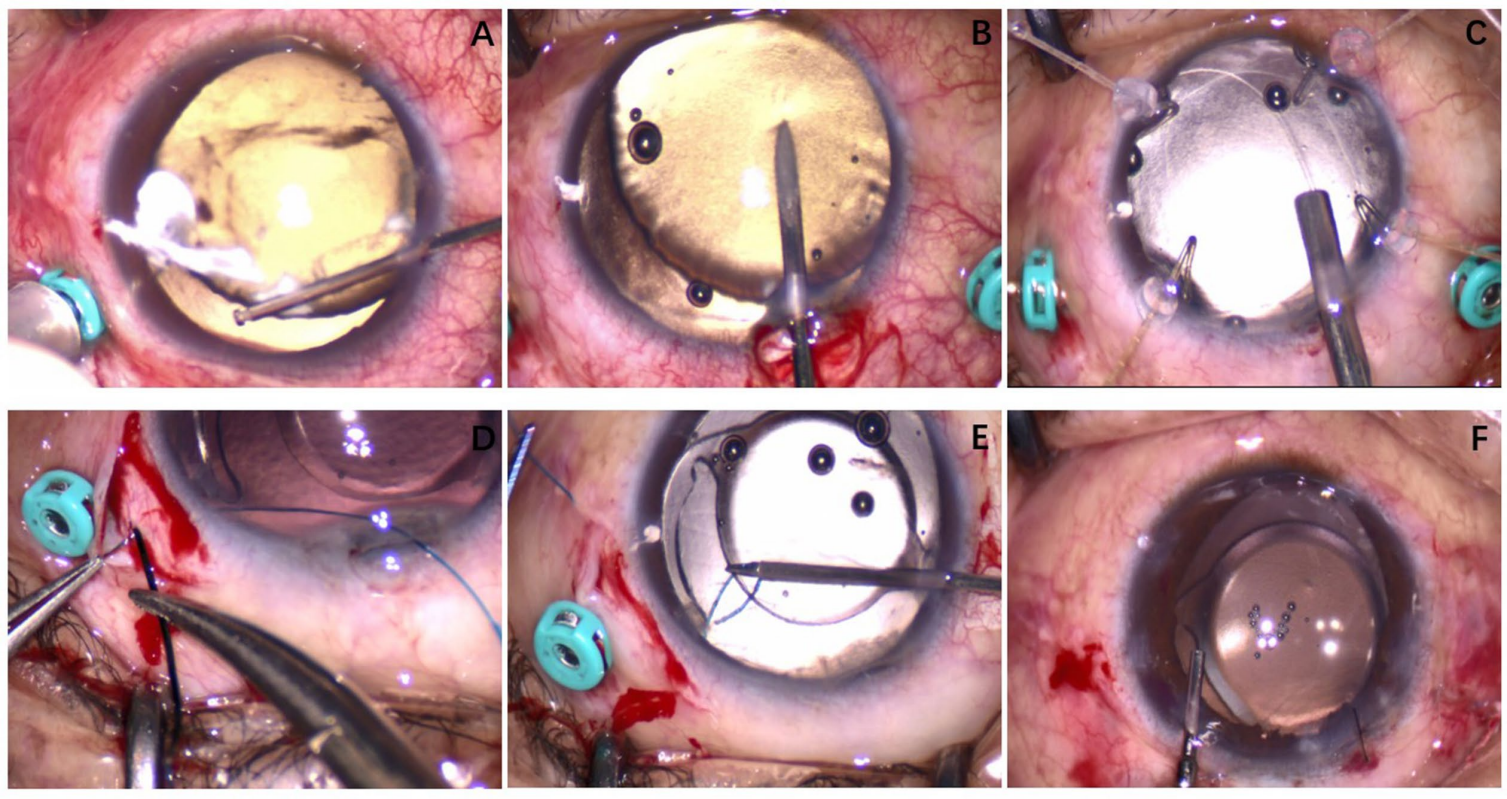
Case 4: A custom-made capsular hook was made using a 6-0 polypropylene suture. **(A)** The patient had zonular dehiscence approximately at the 9 o’clock position, secondary glaucoma, and corneal edema during the operation; anterior chamber vitreous herniation was visible. An anterior chamber vitrectomy was performed. **(B)** The anterior capsule was punctured with a 25G needle. **(C)** After phacoemulsification cataract extraction, a CTR was implanted into the capsular bag. **(D)** The 6-0 polypropylene suture was passed through the sclera. **(E)** The custom-made capsular hook was used to fix the capsulorhexis opening of the lens. **(F)** At the end of the operation, the intraocular lens was centrally positioned.

**Figure 2:**
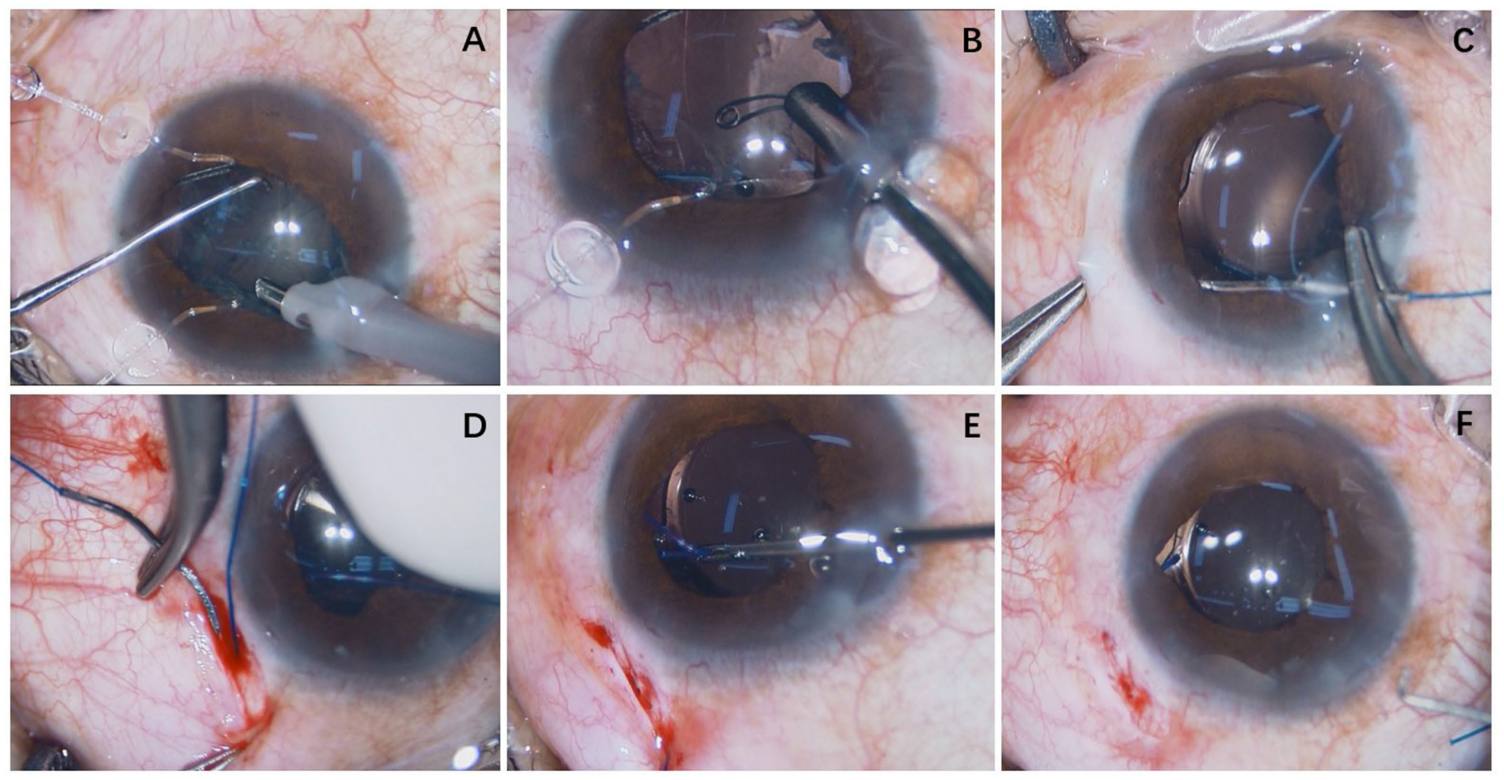
Case 16: A custom-made capsular hook was made using a 5-0 polypropylene suture. **(A)** Phacoemulsification cataract extraction was completed using two capsular hooks. **(B)** The capsular tension ring was implanted into the capsular bag before the irrigation/aspiration step. **(C)** A 5-0 polypropylene suture needle was advanced through the anterior chamber through a clear corneal incision, passing posterior to the iris and anterior to the capsular bag and exiting the eye 2 mm posterior to the limbus. **(D)** The polypropylene suture was passed through the sclera. **(E)** The custom-made capsular hook was used to fix the capsulorhexis opening of the lens. **(F)** At the end of the operation, the intraocular lens was centrally positioned.

The custom-made capsular hook is formed by heat shaping of 5-0 or 6-0 polypropylene sutures (Ethicon, Somerville, New Jersey, United States) into a hook shape. This suture is approved for use in ophthalmic surgeries. The manufacturing method of the custom-made capsular hook is the same as that of Jin et al. (14). The suture is heat-shaped into an acute angle (<15°) by a cautery device, and the head end of the hook is about 1 mm long. All surgeries were performed by the same experienced doctor (NL). Retrobulbar block anesthesia was administered using 2% lidocaine. In cases where vitreous herniation into the anterior chamber was noted, either a limbal or pars plana approach was employed to remove the incarcerated vitreous, thereby preventing intraoperative vitreous disturbance. The 25G vitrectomy system (Constellation Vision, Alcon Laboratories Inc., Fort Worth, Texas, United States) was used. A 3 o’clock clear corneal side incision was made, and sodium hyaluronate gel (HYMIOS, Bloomage Biotechnology Co., Ltd., Shandong, China) was injected into the anterior chamber. The main incision was a 2.8 mm clear corneal incision, positioned to avoid the area with the most severe lens dislocation. The anterior capsule was punctured with a 25G needle, and continuous circular capsulorhexis was completed, with the opening measuring 5 mm. Capsular hooks (CapsuleCare, Madhu Instruments Pvt. Ltd., New Deli, India) were inserted to prevent further extension of the zonular rupture, with 2–4 hooks placed depending on the extent of the rupture. Adequate hydrodissection was performed to facilitate nuclear rotation and reduce intraoperative zonular injury. Phacoemulsification (Centurion, Alcon Laboratories Inc., Fort Worth, Texas, United States) was carried out with an energy of 60%, a negative pressure of 450 mmHg, a bottle height of 80 cmH₂O, and a flow rate of 40 cc/min. The lens cortex was aspirated with a negative pressure of 500 mmHg, a bottle height of 75 cmH₂O, and a flow rate of 35 cc/min. Before removing the phacoemulsification needle and aspiration needle from the anterior chamber, sodium hyaluronate gel was injected from the side incision to maintain the stability of the anterior chamber and prevent further extension of the lens zonular rupture. A injectable CTR (ACPi-11, Bausch & Lomb, Rochester, NY, United States) was implanted into the capsular bag, along with a foldable IOL. The capsular hooks were removed. A 6-0 or 5-0 polypropylene suture with a long needle entered the anterior chamber via a clear corneal incision, passed through the back of the iris and the front of the capsular bag, and penetrated the sclera 2 mm behind the limbus at the area with the most severe lens dislocation. The needle, held by a needle holder, punctured the sclera and traversed it parallel to the limbus for 2–3 mm, exiting through the conjunctiva to form a suture tunnel parallel to the limbus, securing the suture and preventing loosening. While tightening the suture, a 25G retinal forceps (MR-G114T-4, Suzhou Mingren, Suzhou, China) clamped the hook and introduced it into the anterior chamber. The tail of the hook was inserted into the capsulorhexis opening, and the retinal forceps were released. The tension of the suture outside the eye was adjusted to ensure that the hook exerted a pulling force on the anterior capsular opening. One hook can fix the zonular rupture of less than 6 clock hours, two hooks can fix the zonular rupture of approximately 8 clock hours, and for some patients with zonular rupture exceeding 8 clock hours, three hooks can be made for fixation. After adjusting the tension of the hook, the exposed suture was cut off and pushed into the scleral tunnel. The sodium hyaluronate gel in the anterior chamber and capsular bag was aspirated to deepen the anterior chamber and close the incision. For patients who underwent total vitrectomy, the capsular hook fixation was performed after the vitrectomy. The surgical process of the 6-0 polypropylene suture custom-made hook is shown in Figure 1. The surgical process of the 5-0 polypropylene suture custom-made hook is shown in Figure 2.

### 2.3 Ophthalmic examinations

All examinations were conducted by the same experienced ophthalmologist (NL). Pre-operative examination items included slit-lamp microscopy, ultrasound biomicroscopy (UBM-840, Quantel Medical, Clermont-Ferrand, France), B-mode ultrasound, and ophthalmic biometry (IOLMaster 700, Carl Zeiss Meditec AG, Jena, Germany). Intraocular pressure was measured by a non-contact tonometer (NT-530, Nidek Co., Ltd., Gamagori, Japan). Best-corrected visual acuity (BCVA) values, measured by an experienced optometrist, were converted to logMAR. Follow-up visits were conducted at 1, 3, 6, and 12 months after the operation. These visits included evaluating the centration of the intraocular lens, position of the hook, and exposure of sutures under the slit-lamp biomicroscope, as well as optometry, intraocular pressure measurement, and fundus examination. The BCVA at the final follow-up was meticulously recorded and will be employed for in-depth statistical analysis. Post-operative complications were also recorded.

### 2.4 Statistical analysis

The BCVA data of patients before and after surgery were meticulously collated. Since the analysis focuses solely on the single variable of visual acuity, multiple corrections are not required. Both groups’ datasets are complete, with no missing values, allowing for a complete case analysis. To assess normality, the Shapiro–Wilk test was performed on the data.

To assess the normality of the preoperative and postoperative differences, the Shapiro–Wilk test was performed. A *p*-value greater than 0.05 indicated that the differences followed a normal distribution. Additionally, a histogram of the normality test was incorporated to further evaluate whether the data conformed to a normal distribution. If the normal probability plot exhibited a bell-shaped curve (higher in the middle and lower at both ends), it suggested that the data followed a normal distribution. Conversely, a deviation from this pattern implied non-normality.

For variables that followed a normal distribution, data were summarized as mean ± standard deviation (SD). In contrast, for variables that did not conform to a normal distribution, data were reported as median and interquartile range (IQR) to better represent the central tendency and variability of the dataset.

For normally distributed differences, a paired sample *t*-test was conducted for statistical analysis, Cohen’s *d* was reported to quantify the effect size. However, if the Shapiro–Wilk test yielded a significant result (*p* < 0.05), indicating that the differences deviated from a normal distribution, a paired sample Wilcoxon signed-rank test was applied instead, with the Hodges–Lehmann median difference reported as a measure of effect size. To assess the uncertainty of the Hodges–Lehmann median difference, bootstrapping was performed using the boot package in R version 4.2.2 (The R Foundation, Vienna, Austria). The original difference data were resampled 1,000 times, and the 95% confidence interval for the Hodges–Lehmann median difference was computed.

A *p*-value less than 0.05 was considered statistically significant. Statistical analyses were conducted using SPSS version 25.0 (IBM Corp., Armonk, NY, United States) and R version 4.2.2 (The R Foundation, Vienna, Austria).

## 3 Results

This technique was applied to 16 patients (16 eyes) (Table 1). Seven patients (cases 1, 2, 3, 4, 6, 10, and 16) received capsular hooks made of 5-0 polypropylene sutures, whereas the remaining patients had capsular hooks made of 6-0 polypropylene sutures. Seven patients (cases 1, 2, 4, 9, 11, 13, and 15) had secondary glaucoma before surgery, with intraocular pressure normalizing post-treatment. Ten patients (cases 4, 5, 6, 7, 9, 10, 11, 12, 13, and 15) had vitreous herniation in the anterior chamber preoperatively and underwent anterior vitrectomy via the limbal or pars plana approach. One patient (case 8) had vitreous hemorrhage before surgery and underwent total vitrectomy through the pars plana approach. Ten patients received one hook, five patients with zonular rupture of more than 6 clock hours received two hooks, and one patient (case 13) with 10 clock hours of zonular dehiscence received three hooks. One patient (case 10) had puncture site bleeding during the second needle fixation. Due to the large amount of bleeding, blood dispersed into the vitreous cavity requiring total vitrectomy through the pars plana approach during the operation. No other intraoperative complications occurred. Post-operative follow-up was 6–30 months, with an average of 10 ± 5.9 months.

**Table 1:** Demographic and clinical data of eyes with subluxated lens.

Case	Sex	Age	Eye	Zonular defect (clock hours)	BCVA (logMAR)		IOP (mmHg)		Follow-up (months)	Management	Hooks used for fixation	Complications		
					Preoperative	Postoperative	Preoperative	Postoperative				Preoperative	Intraoperative	Postoperative
1	F	66	L	8	0.7	0.2	36	15	12	Phaco + CTR + capsular hook + goniosynechialysis	2	SG	None	None
2	M	56	R	6	2.1	0.5	42	18	30	Phaco + CTR + capsular hook + goniosynechialysis	1	SG	None	RRD
3	M	71	L	5	2.4	0.3	15	14	8	Phaco + CTR + capsular hook	1	None	None	None
4	M	49	L	9	2.3	0.2	Over	19	6	Phaco + CTR + capsular hook + two-point pars plana vitrectomy	2	SG, vitreous herniation	none	none
5	M	54	R	5	0.8	0.1	13	14	10	Phaco + CTR + capsular hook + two-point pars plana vitrectomy	1	Vitreous herniation	None	None
6	F	70	R	8	0.7	0.1	18	16	12	Phaco + CTR + capsular hook + one-point pars plana vitrectomy	2	Vitreous herniation	None	None
7	M	68	L	6	2.4	0.5	16	17	6	Phaco + CTR + capsular hook + limbal vitrectomy	1	Vitreous herniation	None	None
8	M	39	R	6	2.5	0.5	15	16	10	Phaco + CTR + capsular hook + pars plana vitrectomy	1	VH	None	None
9	M	53	L	5	3.0	0.8	Over	19	8	Phaco + CTR + capsular hook + limbal vitrectomy	1	SG, vitreous herniation	None	None
10	M	74	L	7	0.6	0.4	14	15	6	Phaco + CTR + capsular hook + pars plana vitrectomy	2	Vitreous herniation	VH	None
11	F	76	R	7	2.5	0.2	32	15	12	Phaco + CTR + capsular hook + one-point vitrectomy	2	Vitreous herniation	None	None
12	M	37	R	5	1.1	0	13	15	6	Phaco + CTR + capsular hook	1	Vitreous herniation	None	None
13	M	69	L	10	1.7	0.3	45	17	10	Phaco + CTR + capsular hook + limbal vitrectomy	3	SG, vitreous herniation	None	None
14	F	74	R	5	2.4	0.3	38	14	12	Phaco + CTR + capsular hook + goniosynechialysis	1	SG	None	None
15	F	61	R	6	0.4	0	11	15	6	Phaco + CTR + capsular hook + two-point pours plana vitrectomy	1	SG, vitreous herniation	None	None
16	M	55	L	7	2.2	0.1	18	17	6	Phaco + CTR + capsular hook	1	None	None	None

F, female; M, male; BCVA, best-corrected visual acuity; IOP, intraocular pressure; SG, secondary glaucoma; VH, vitreous hemorrhage; RRD, rhegmatogenous retinal detachment.

**Figure 3:**
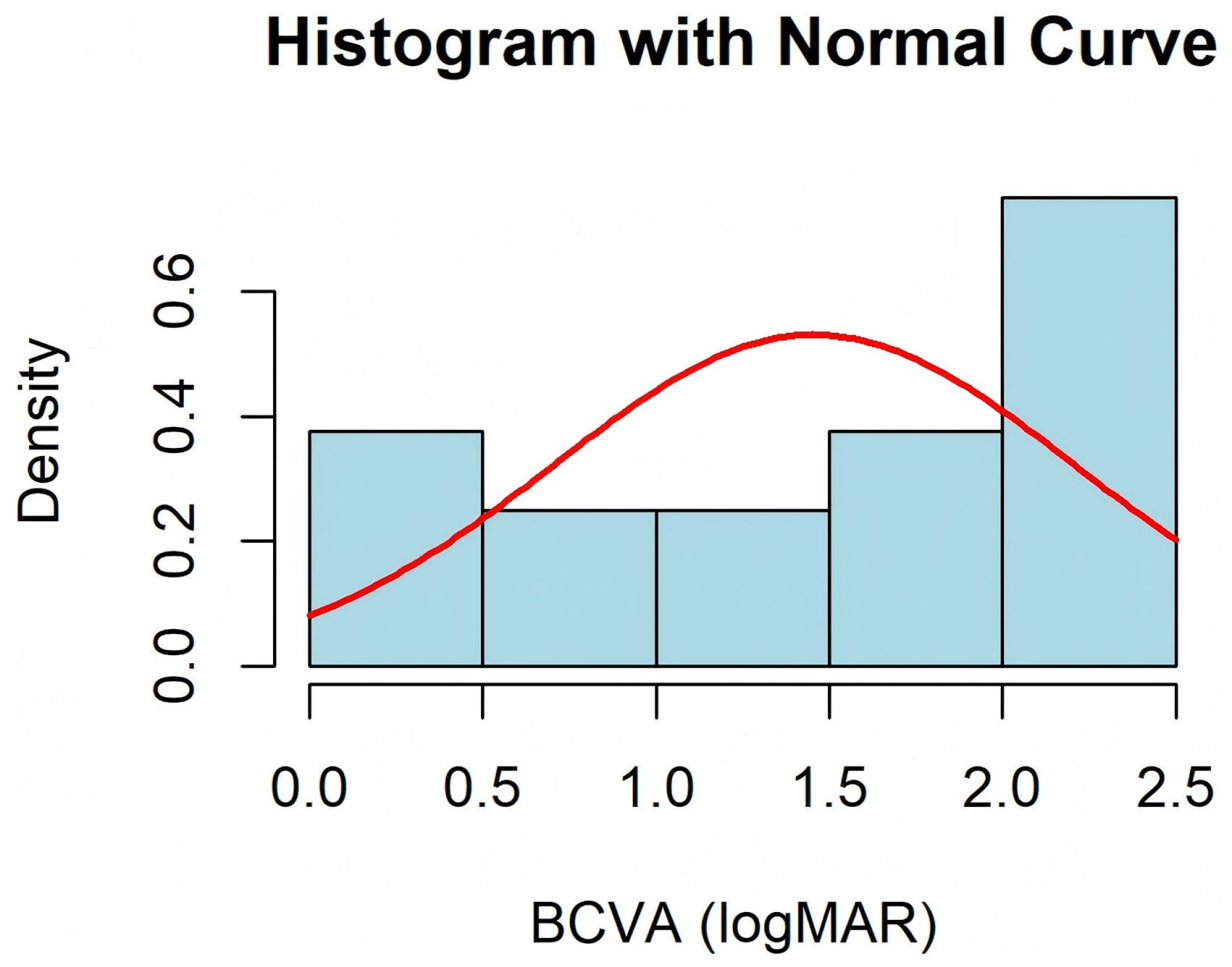
Histogram plot of BCVA.

**Figure 4:**
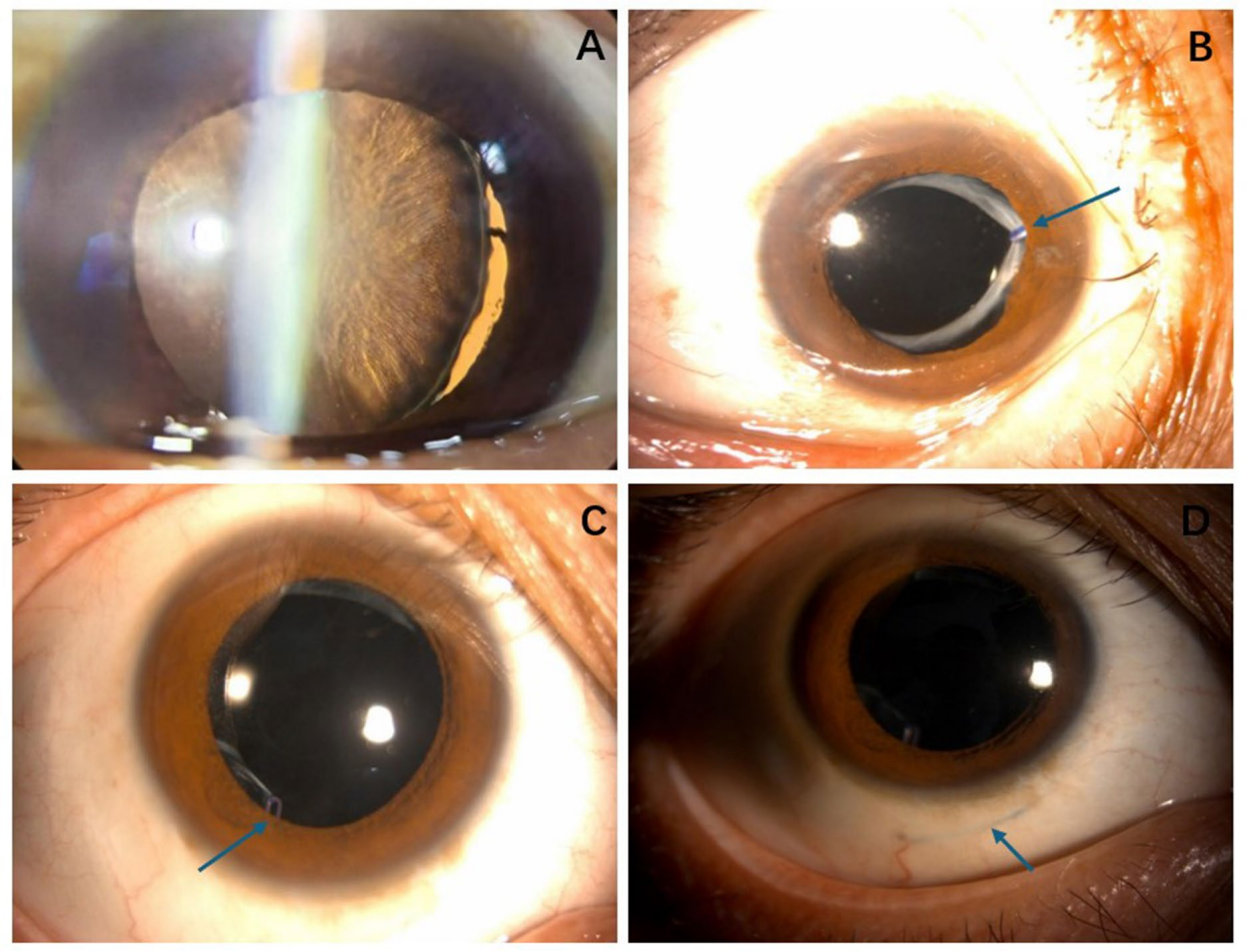
**(A)** Case 16: Lens zonule rupture occurred at approximately the 6 o’clock position after trauma. **(B)** Case 16: Six months after surgery, the intraocular lens was properly centered, and the capsular tension ring remained securely fixed (arrow). **(C)** Case 12: One year after the operation, the intraocular lens remained centered, and the tension ring could be seen at the lower anterior capsular opening (arrow). **(D)** Case 12: One year after the surgery, the 6-0 polypropylene sutures were located within the scleral lamellae, with no signs of loosening or exposure (arrow).

The Shapiro–Wilk normality test indicated that the differences in BCVA did not follow a normal distribution (*W* = 0.855, *p* = 0.016). This finding was further supported by the histogram, which suggested a skewed distribution (Figure 3). Consequently, a paired sample Wilcoxon signed-rank test was performed, revealing a significant improvement in BCVA from a preoperative median of 2.15 (1.67) logMAR to a postoperative median of 0.25 (0.38) logMAR (*Z* = 3.519, *p* < 0.001). The Hodges–Lehmann median difference was −1.75 (95% CI: −2.10 to −1.40), suggesting that the median postoperative BCVA was 1.75 logMAR units lower than preoperative values. After surgery, all patients maintained well-centered IOLs, with no complications such as dislocation of the IOL-capsular bag complex, hook loosening, suture exposure (Figure 4), vitreous hemorrhage, or cystoid macular edema. One patient (case 2) had rhegmatogenous retinal detachment 2 years after surgery. After pars plana vitrectomy, the retina was reattached, and the final visual acuity remained stable.

## 4 Discussion

Lens subluxation is a common clinical condition. In China, traditional surgical approaches for lens dislocation, particularly in cases of moderate to severe subluxation, often involve removing the dislocated lens and performing one-stage or two-stage suspension of the IOL. These approaches, however, do not preserve the lens zonules and may lead to vitreous disturbance during the procedure. The probability of post-operative vitreoretinal complications such as vitreous hemorrhage and retinal detachment is high. Furthermore, these procedures can cause considerable surgical trauma, resulting in poor visual prognosis and adverse physical, psychological, and economic effects for the patient.

However, advancements in technology, surgical methods, material innovation, and conceptual developments have led to a paradigm shift in the management of moderate and severe subluxation. Current approaches emphasize the preservation of the capsular bag and restoration of the lens zonules whenever feasible. Surgical success relies on the use of long-term capsular fixation devices during and after the operation. Common intraoperative capsular stabilization devices include iris and capsular hooks. Due to their structural advantages and optimal angles, capsular hooks offer superior intraoperative support in stabilizing the capsule and preventing the extension of zonular rupture. Consequently, they are widely used in lens dislocation surgery. Proper post-operative capsular fixation is the key to maintaining long-term capsular stability and centering the IOL.

Various approaches for managing lens dislocation have been employed domestically and internationally. In 1998, Professor Robert Cionni’s team reported the treatment of four patients with lens subluxation using the MCTR, achieving favorable outcomes (15). Many studies have also reported that the treatment of subluxation of the lens using the MCTR has good post-operative results (16–18). Doctors represented by Professor Ike Ahmed in Canada choose the capsular tension segment (CTS) for long-term capsular fixation (19, 20). An Israeli doctor, Assia, used the capsular anchor for capsular fixation. He first conducted experiments on rabbit eyes, with a good fixation effect (21). Subsequently, Professor Yokrat Ton reported the application of the capsular anchor in patients with subluxation of the lens and dislocation of the IOL-capsular bag complex. The long-term capsular fixation effect was good, and the IOL was centered (22). Japanese scholars, Asano et al. (23), reported the use of a capsular hook made of 5-0 polypropylene sutures for capsular fixation, with patients followed up for 2 years. The position of the IOL was good, and the capsular bag complex remained stable for a long time. This method evolved from the early self-made capsular hook of 5-0 polypropylene sutures by this team and was improved to achieve good results (24). Polish doctors, Krix-Jachym et al. (25), reported that the use of iris hooks for long-term capsular fixation also achieved good results. Professor Karadag et al. (26) also reported applying this surgical method.

Professor Jiang Yongxiang was an early adopter of the MCTR for lens dislocation treatment in China, achieving excellent results. It was also the best treatment method. However, MCTR is currently out of stock in China, preventing many patients from benefiting from this successful treatment option. Professor Haiying Jin reported the use of polypropylene sutures to make iris hooks in 2018 (27). In 2019, he reported the use of 5-0 polypropylene sutures, heat-shaped into capsular hooks, to stabilize the dislocated intraocular lens-capsular bag complex. Later, he applied this technique to 10 patients with lens dislocation and achieved good results (28). Later, this method was quickly adopted throughout China. In the absence of commercial capsular fixation devices such as MCTR, CTS, and capsular anchors, it effectively solved the long-term fixation problem of the capsular bag. Moreover, this approach does not entail any extra financial strain on patients, ensuring that the treatment remains cost-accessible and alleviating potential economic concerns during the medical process. Professor Jiang Yongxiang’s team also reported the treatment of 148 eyes of patients with Marfan syndrome using CTR combined with implantable capsular hooks. Compared with the MCTR, the CTR-CH procedure was a feasible, safe, and efficient approach for managing EL in patients with MFS (29).

According to the safety and effectiveness data provided above, our team adopted a surgical approach for traumatic crystalline lens subluxation, achieving favorable outcomes. This approach involves phacoemulsification cataract extraction, using capsular hooks, CTR, and IOL implantation in the capsular bag combined with anterior capsular fixation with custom-made capsular hooks. However, most studies primarily examine congenital lens dislocation, particularly in conditions like Marfan and Marchesani syndromes, with limited focus on the surgical management of traumatic lens dislocation. Blunt ocular trauma is a major cause of traumatic subluxation of the lens. Due to external forces, the rupture or relaxation of the lens zonules occurs, resulting in an abnormal lens position. This condition can also result in cataract formation and visual impairment. Traumatic lens dislocation is distinct from other subluxation types in that it may involve injuries other than zonular injuries, such as angle recession, iris root rupture, traumatic mydriasis, hyphema, and vitreous hemorrhage. Zonular rupture can cause damage to the ciliary epithelium and ora serrata, potentially resulting in vitreous hemorrhage and retinal detachment. Trauma-induced vitreous anterior membrane injury can lead to vitreous incarceration in the anterior chamber and forward movement of the lens-iris diaphragm due to lens dislocation. The consequent angle closure and angle recession can cause secondary glaucoma. Elevated pre-operative intraocular pressure and corneal edema may occur in some patients as a result of vitreous contact with the corneal endothelium. Surgical intervention is necessary to address these complications. Unlike other lens dislocation surgeries, the removal of incarcerated vitreous from the anterior chamber is required to facilitate the procedure and prevent interference during the procedure. In some cases, anterior vitreous removal and indentation may be necessary to examine the ora serrata and ciliary epithelium for possible lacerations. For patients with vitreous hemorrhage, vitrectomy is often required in one stage, which brings more challenges to our surgery.

Of the 16 patients in our study, seven had secondary glaucoma. All patients had elevated intraocular pressure preoperatively, and conservative treatments showed minimal effect. Corneal edema was pronounced, which hindered surgical visibility. Thus, we used a 25-gauge vitrectomy system for illumination, which enabled us to perform continuous circular capsulorhexis and resolve the issue of the absent red reflex. The support and elevation provided by the light guide fiber also helped prevent lens sinking, limiting further lens dislocation. Anterior vitrectomy was performed in cases 4, 9, 11, and 13. Cases 1, 2, and 15 underwent goniosynechialysis under gonioscopy. The above surgical operations effectively resolved pupil block and separated adherent or narrow angles, thus addressing the glaucoma. The post-operative intraocular pressure remained stable for a long time, confirming the success of our approach to secondary glaucoma. Cases 5, 6, 7, and 15 had vitreous incarceration in the anterior chamber. However, the vitreous incarceration and lens dislocation did not cause pupil block, angle stenosis, or adhesions, so intraocular pressure remained stable. Anterior vitrectomy was performed in these cases to resolve the vitreous incarceration. Intraoperative indentation did not reveal any lacerations in the ciliary epithelium or ora serrata. Case 8, which had a large amount of pre-operative vitreous hemorrhage, underwent combined anterior and posterior segment surgery. This approach addressed the lens dislocation and managed the associated vitreoretinal conditions, leading to a favorable outcome.

In case 10, vitreous hemorrhage occurred when the second needle of the 5-0 suture punctured the tissue. This was likely due to the suture needle damaging blood vessels in the pars plana of the ciliary body. Although we avoided puncturing at the 3 and 9 o’clock positions, vitreous hemorrhage still occurred. This indicates that vitreous hemorrhage is a common complication during suture needle puncture, but the incidence is not high. Due to timely vitrectomy, the visual prognosis was not considerably impacted by the hemorrhage. Case 2 developed retinal detachment 2 years post-surgery. We did not perform anterior vitrectomy or check the ora serrata during the operation, with the pre-operative ultrasound biomicroscopy (UBM) and B-ultrasound showing abnormalities in the ciliary epithelium and ora serrata. Thus, we concluded a tractional horseshoe-shaped hole caused by post-operative vitreous detachment contributed to the retinal detachment. Intraoperative exploration confirmed this, revealing no vitreous proliferation or traction at the suture site. After vitrectomy, the retina was reattached and visual acuity remained stable. This underscores the potential risk of retinal detachment following surgery, which necessitates thorough pre-operative discussions with patients. Case 9 had an iris root rupture, which was successfully repaired using the modified sewing machine method in a single-stage procedure, resulting in good post-operative iris morphology. Case 12 was a patient with high myopia. Pre-operative UBM showed zonular laxity, but the lens position remained centered without significant deviation. During surgery, a CTR and foldable IOL were implanted in the capsular bag. When the viscoelastic agent was aspirated, a zonular rupture occurred at approximately the 6 o’clock position. This was likely due to the zonular disease associated with high myopia, liquefied vitreous, and elevated intraoperative perfusion pressure. To address the rupture, a 6-0 polypropylene suture was used to secure the anterior capsular opening at the 6 o’clock position. After the operation, the IOL was centered, with a well-fixed capsular bag. This case illustrates that self-made capsular hooks can be an effective solution for centering the IOL in the presence of zonular rupture caused by intraoperative injury.

Through the surgical management of 16 patients with traumatic lens subluxation, we have identified several key principles. First, detailed preoperative evaluations, such as anterior segment photography, intraocular pressure measurement, ocular B-ultrasound, and UBM examination (30), are extremely necessary. Intraoperatively, careful vitreous management, especially when dealing with incarcerated vitreous in the anterior chamber or vitreoretinal complications such as vitreous hemorrhage, is crucial. The main incision should avoid the area with the most severe lens dislocation. Good continuous circular capsulorhexis is a crucial step in preserving the capsular bag. Due to the lack of zonular traction and poor capsular tension, a 25 g needle can be used to puncture the anterior capsule and create a flap. To prevent capsule tearing or complications with hook placement during or after the operation, the size of the capsulorhexis opening should not be too large, which could result in poor fixation. Preventing further zonular injury during the procedure requires maintaining anterior chamber stability. Before making the main incision, sodium hyaluronate gel should be injected to maintain the stability of the anterior chamber. According to the range of lens dislocation of the patient, an appropriate number of capsular stabilization devices should be selected, with the capsular hook being highly recommended. Adequate hydrodissection is important to reduce resistance during nuclear rotation, which helps prevent the worsening of zonular rupture. Perfusion pressure should be reduced during the operation, and sodium hyaluronate gel should be injected into the anterior chamber before withdrawing the phacoemulsification and aspiration needles. When performing CTR implantation, if zonular support is insufficient, the CTR should be placed in the capsular bag before cortex aspiration. However, if the zonular support is acceptable, the CTR can be implanted after cortex aspiration. The maximum support of CTR should be directed toward the area with the most severe dislocation. This facilitates the effective expansion of the capsular bag and ensures full outward expansion of the equatorial portion. Therefore, CTR implantation is an essential material for the long-term stability of the IOL-capsular bag complex. When selecting an IOL, foldable lenses should be prioritized for easier positioning, and one-piece non-plate IOLs are particularly recommended. After implanting the IOL, the capsular hook can be removed. 5-0 and 6-0 polypropylene sutures can be heat-shaped into hook shapes, with no difference in the long-term fixation effect of the capsular bag. The hook should be formed at an acute angle, with the head retaining a minimum length of 1 mm to prevent loosening after the procedure. Once the suture exits the eye, it should pass through the sclera before the hook is implanted. The 6-0 polypropylene suture, being thinner and having a sharper needle, is recommended for creating the capsular hook, especially when considering the difficulty of passing through the sclera. For patients who need intraoperative indentation or vitrectomy, it is crucial to secure the hook after properly managing the vitreous to prevent the loosening of the hook from the capsular opening. If loosening occurs, the hook can be repositioned by performing indentation of the pars plana under microscopic guidance. In the case of iris root rupture, repair should be attempted during the procedure. Some patients in the 16-patient cohort experienced mydriasis, with the largest dilation reaching 6 mm. Pupiloplasty was not performed during the operation, and photophobia was not detected after the operation. Therefore, for patients with traumatic mydriasis with photophobia, pupiloplasty can be completed in the second stage. Regular post-operative follow-up and rational drug use are also important.

However, our study also has certain limitations. The cohort size was relatively modest, which may not comprehensively represent the full spectrum of clinical scenarios. Moreover, the follow-up duration for a subset of patients was inadequate. This shortcoming restricts our ability to draw definitive conclusions regarding the long-term stability and fixation effectiveness of the capsular bag, which undoubtedly necessitates extended and more in - depth observation. Another notable limitation lies in the singularity of the surgical approach we utilized. In the realm of capsular bag fixation, there exist several well-established techniques, such as the MCTR and CTS. Regrettably, we did not perform a side-by-side comparison between our method and these alternative procedures. Such a comparative analysis would have been invaluable in discerning the relative advantages and disadvantages of each approach, thereby providing more robust evidence-based guidance for clinical practice.

## 5 Conclusion

According to long-term follow-up data, heat-shaped 5-0 or 6-0 polypropylene sutures can be used in patients with traumatic crystalline lens subluxation to fix the anterior capsular opening with custom-made capsular hooks, thereby achieving long-term stability of the capsular bag. This technique maintains the centering of the IOL postoperatively, showing effectiveness and safety. Moreover, this approach does not entail any extra financial strain on patients, and it holds potential for broad clinical applications.

## Data Availability

The datasets presented in this study can be found in online repositories. The names of the repository/repositories and accession number(s) can be found in the article/supplementary material.
